# Supplementary material for: Evaluating lncRNA Expression Patterns during HIV-1 Treatment Interruption
Source: Int J Mol Sci. 2023 Jan 5;24(2):1031. doi: 10.3390/ijms24021031 (PMC9866393; doi:10.3390/ijms24021031)
Supplement: Supplementary file 1 [file ijms-24-01031-s001.zip › ijms-2069543-supplementary.pdf]

Supplemental figures and tables

Table S1: Primers used for quantitative PCR

Gene	Forward primer (5'-3')	Reverse primer (5'-3')
APOBEC3G	TTCCTTCCTGGGCATGGAGT	TACAGGTCTTTGCGGATGTC
ACTB	TTCCTTCCTGGGCATGGAGT	TACAGGTCTTTGCGGATGTC
GAPDH	AGCCTCAAGATCATCAGCAATGCC	TGTGGTCATGAGTCCTTCCACGAT
GAS5	TCTTGCCTCACCCAAGCTAGAG	TTGTGCCATGAGACTCCATCAG
HEAL	GTATCTCACCGTCCCAGAATG	GAGATGAACCCTCTGCTTGTC
IFIT1	GATCTCAGAGGAGCCTGGCTAA	TGATCATCACCATTTGTACTCA
MALAT1	AGTTGGGATCAAGTGGATTG	CTCCCAGATGAAATGAAGCA
MX1	CTGTAAATCTCTGCCCCTGTTAG	TCGTGTCGGAGTCTGGTAAAC
MX2	GAAACAGGAGCCAACCAA	GAGTCGATGAGGTCAATGC
NEAT1 transcript 1	CTTCCTCCCTTTAACTTATCCATTCAC	CTCTTCCTCCACCATTACCAACAATAC
NEAT1 transcript 2	CAGTTAGTTTATCAGTTCCATCCA	GTTGTTGTCGTCACCTTTCAACTCT
NRON	ACGTTCCTTAATGTACGCCTTTGC	TTGGCCGTGTCCTGAGTCCTT
SAMHD1	PrimePCRTM PreAmp for SYBR Green	Assay: SAMHD1, Human (Bio-Rad)
SLFN11	TGTTCCAGGCCTTTCTTGG	GATGACAGACACAGATCCAGAT
Tetherin/BST2	CAAGCTCCTCCACTTTCTTT	CCCATCTCCTGCAACAAG
TRIM5	CTCGAAACACTCTCCTTTGA	GTGTGGATGGCGTCATAA
YWHAZ	ACTTTTGGTACATTGTGGCTTCAA	CCGCCAGGACAAACCAGTAT

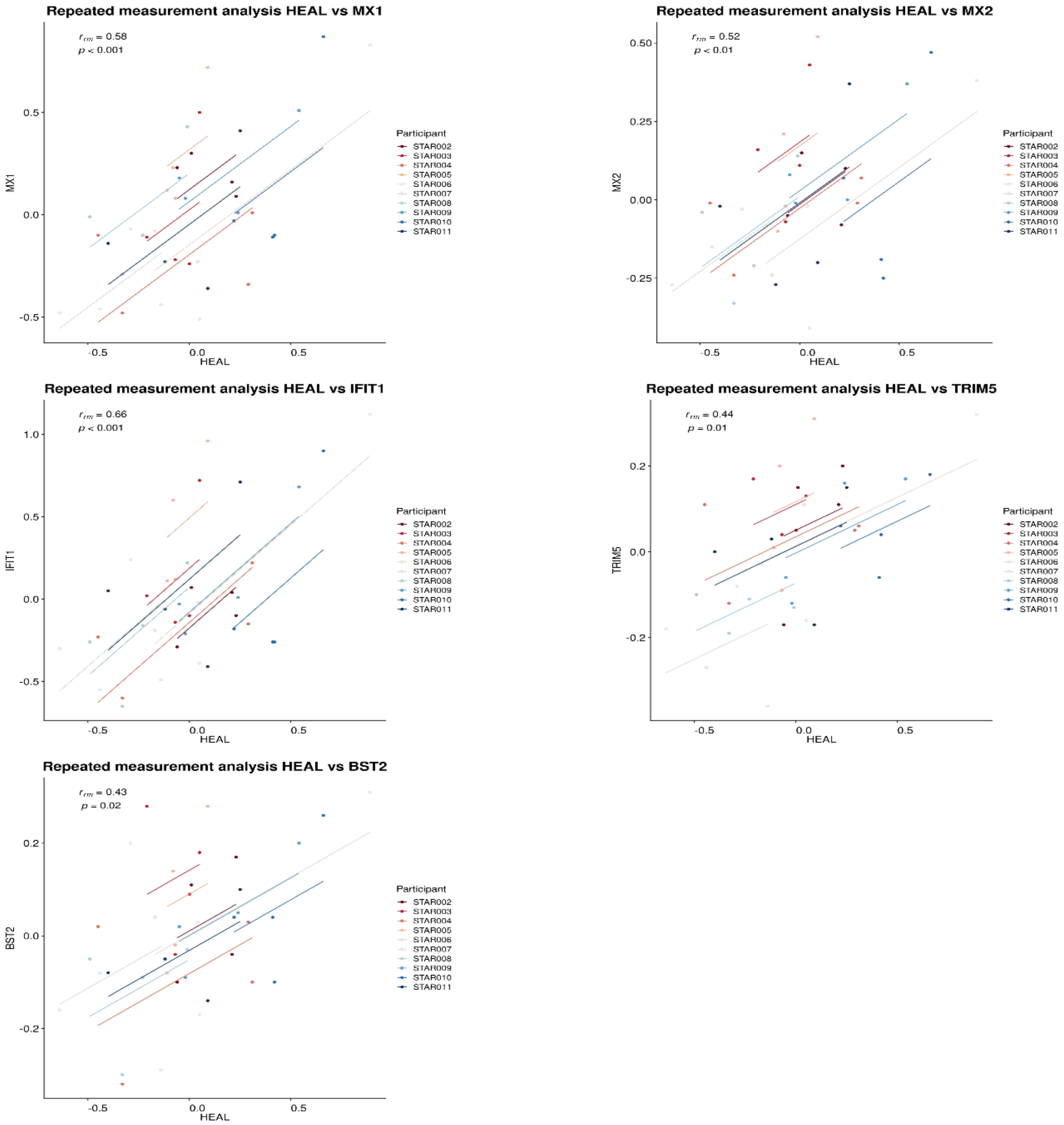

Figure S1: Repeated measurement analysis. Repeated measurement analysis was performed between the expression of HEAL and MX1, MX2, IFIT1, TRIM5 and BST2. Expression levels on the y and x-axis are given in Log scale. r_{rm}: repeated measurement r.

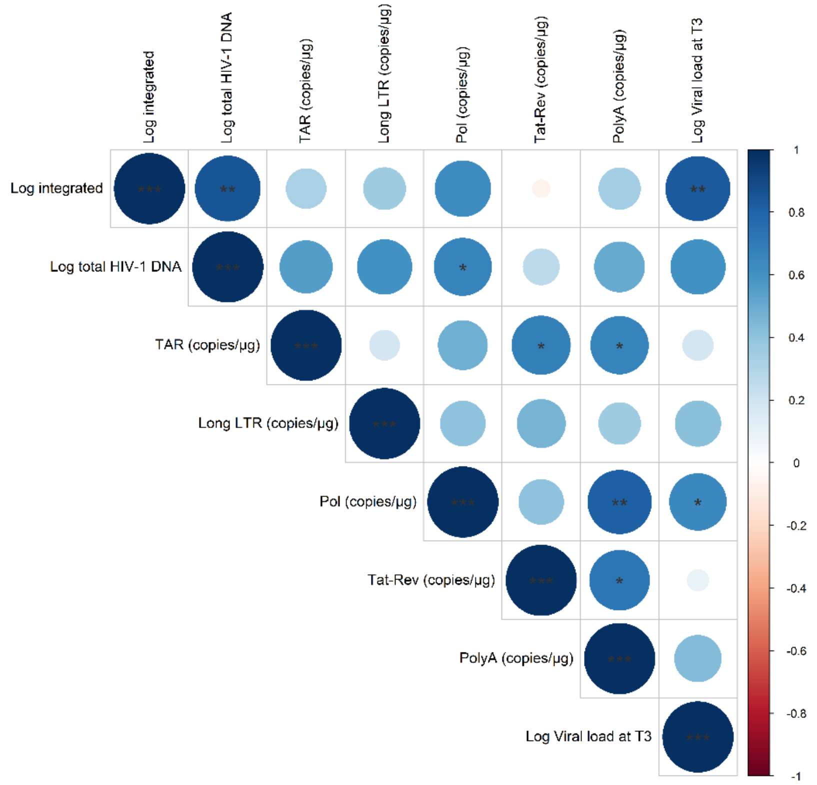

Figure S2: Correlation plot indicates the correlation between the viral load at T3 and HIV-1 viral DNA and RNA markers in participants undergoing ATI. Spearman correlations were calculated including all participants. Positive and negative correlations are depicted in blue and red, respectively. Significant correlations are indicated with asterisks (p < 0.05: *, p < 0.01: ** and p < 0.001: ***).

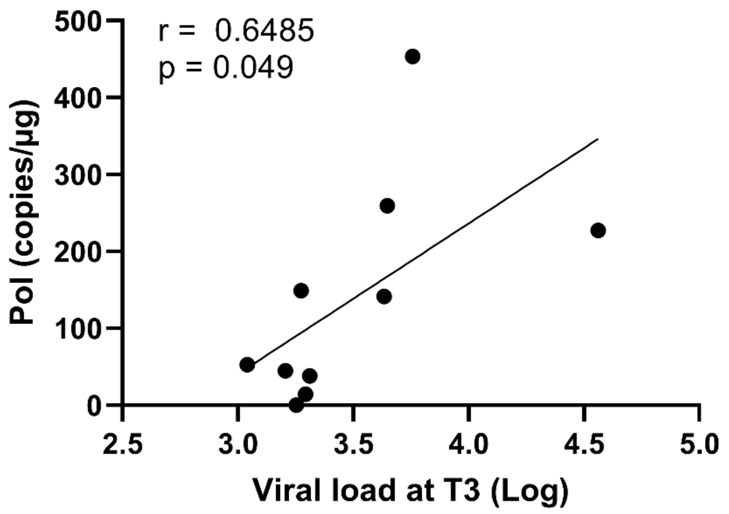

Figure S3: Spearman correlation plot of the expression of Pol RNA and the viral load at T3 of the ATI, including all patients. The Spearman r is reported as well as the p-value.

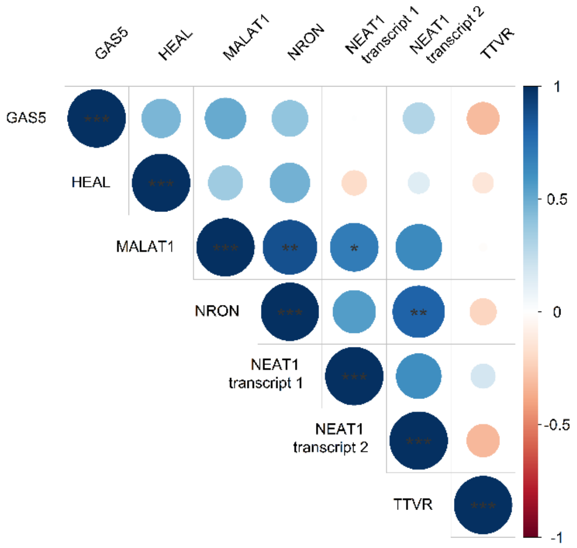

Figure S4: Correlation plot indicates the correlation between the expression of the lncRNAs at T2 and the time to viral rebound. Spearman correlations were calculated including all participants. Positive and negative correlations are depicted in blue and red, respectively. Significant correlations are indicated with asterisks (p < 0.05: *, p < 0.01: ** and p < 0.001: ***).

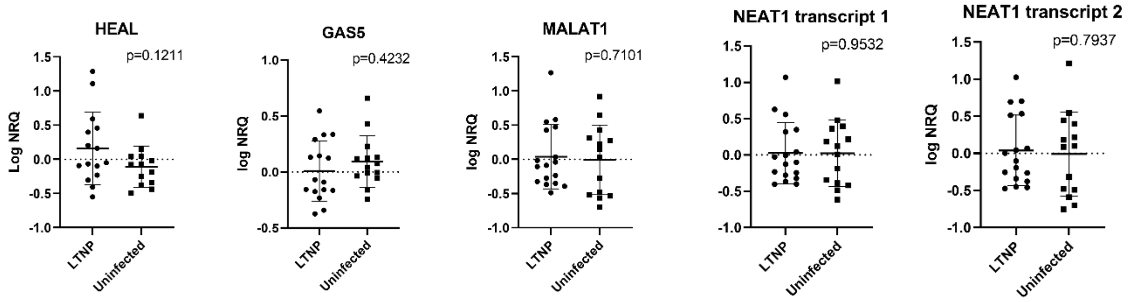

Figure S5: expression levels of HEAL, GAS5, MALAT1 and NEAT1 transcript 1 & 2 in LTNP (n = 17)_and uninfected (n = 14) individuals. Expression is given in Log NRQ and the p-value between the two groups is given.
